# Supplementary material for: ‘‘Ask the way from those who have walked it before”—Grandmothers’ roles in health-related decision making and HIV pre-exposure prophylaxis use among pregnant and breastfeeding women in sub-Saharan Africa
Source: PLoS One. 2022 Sep 1;17(9):e0271684. doi: 10.1371/journal.pone.0271684 (PMC9436035; doi:10.1371/journal.pone.0271684)
Supplement: S1 File — (PDF) [file pone.0271684.s001.pdf]

MTN-041

Grandmother Focus Group Discussion (FGD) Topic Guide

INSTRUCTIONS for the Facilitator: How to use the FGD Guide

1. There are two levels of questions:

    - Primary discussion questions: appear in **bold** text and represent all the topics you will need to cover by the end of the interview. The questions are written to ensure some consistency across FGDs but you are not required to read them verbatim. You may adapt the questions and/or ask them in a different order, depending on how the discussion develops.
    - Probing topics: are indicated with a bullet. If you find that the participants provide little information in response to the primary question, these probing topics may be used to encourage further discussion. You are not required to cover every topic listed.

2. *Instructions/suggestions to facilitator are in italics and [brackets].*

3. P = pregnancy & BF = breastfeeding, which are used throughout the guide

4. The FGD guide is not meant to be used to take notes. Rather, you should use the separate notes form, where you will also insert your initials, the PTIDs of all participants, as well as the date, start and end time of the discussion.

5. Should there be need to conduct an in-depth interview (IDI) in lieu of an FGD, per approval from the management team, this FGD guide can be used for an in-depth interview (IDI) guide.

Before starting the FGD, ensure that all participants have provided written informed consent.

____
Staff Initials / Date

A. Introduction

[*Facilitator should explain the following points – PLEASE DO NOT READ VERBATIM:*]

- Purpose of FGD:
  - Discuss practices and taboos during pregnancy and breastfeeding
  - Get your opinions about your daughter or daughter-in-law using vaginal products and oral medications during pregnancy and breastfeeding
  - Understand what may influence mother or mother-in-law's support of pregnant and breastfeeding women's use of two HIV prevention methods: a vaginal ring and oral tablets
- Affirm to the participants that they are the experts and that all answers are valid: no right and wrong
- Invite differing opinions
- Remind participants that the discussion is confidential, so personal information won't be shared outside of the study
- Tell participants to use pseudonyms for themselves and anyone else they mention so as to preserve confidentiality
- Participants should identify themselves with their pseudonym each time prior to raising a point and speak one at a time so that the audio recorder can capture everything
- Remind participants to keep cellphones silent throughout discussion to avoid disruption of audio recording
- *[Turn audio recorder on]* Ask participants to confirm for audio recorder that they agree to participate in FGD *[be sure to get a verbal okay from all members before continuing]*

B. Health, HIV Worry, and Decision Making while Daughters/Daughters-in-law are Pregnant or Breastfeeding

Purpose: To ask about health practices and restrictions for pregnant and breastfeeding women including HIV risk and worry.

1. **What do mothers/mothers-in-law in your community do to ensure their daughters/daughters-in-law stay healthy while pregnant?**

Possible probing topics:

- *KEY PROBE*: How about while breastfeeding?
- *KEY PROBE*: What restrictions/taboos are there for P and BF women in your community?
- What practices or restrictions are for the purpose of the mom's health versus the baby's health?
- What changes do women make to their diets or activities? Why?

Purpose: To understand who makes or influences health-related decisions for women while pregnant or breastfeeding.

2. **Who makes decisions about what a woman should or should not do while pregnant?**

Possible probing topics:

- *KEY PROBE*: How about while breastfeeding?
- *KEY PROBE*: How involved are the mothers/mothers-in law in making decisions about what your daughter/daughter-in-law should/should not do while pregnant or breastfeeding?
- *KEY PROBE*: How do you discuss what you think your daughters/daughters-in-law should or shouldn't do with her?
- From where do mothers/mothers-in-law get their views about P & BF?
- From whom do women ask for advice about P & BF?

3. **What fears or concerns do mothers/mothers-in law in your community have about their daughters'/daughters-in-law pregnancies?**

Possible probing topics:

- *KEY PROBE*: How about fears or concerns while breastfeeding?
- Where do you think these concerns/ fears come from?
- What do mothers/mothers-in law do to alleviate those concerns?
- How do you discuss these concerns with your daughters/daughters-in-law?

4. **While your daughter/daughter-in-law is pregnant, from whom and for what do you encourage her to seek care?** (medical doctor, traditional birth attendant, traditional healer, chief, religious leader, family members, or other key influencer)

Possible probing topics:

- *KEY PROBE*: How about while breastfeeding?
- *KEY PROBE*: If she was given conflicting guidance, whose recommendation would you urge her to follow? Why?

5. **What kinds of products do women take, eat, rub on their body or insert to prepare the birth canal?**

Possible probing topics:

- *KEY PROBE:* What about while breastfeeding?
- *KEY PROBE*: How have you been involved with helping your [daughter/daughter-in-law] prepare for birth or breastfeeding?
- *KEY PROBE*: What kind of guidance do you provide to your daughter/daughter-in-law about medication use during pregnancy?
- When is it okay or not okay to take medications, herbs or products while P or BF?
  - From whom did you learn about these "rules"?

6. **How much do you think pregnant women are at risk of getting HIV?**

Possible probing topics:

- *KEY PROBE*: What about while breastfeeding?
- How important is it for a woman to protect herself from HIV before, during and after pregnancy? Why so?

C. Sexual Activity and Vaginal Practices while Pregnant/Breastfeeding

Purpose: To understand changes in sexual activity and vaginal practices while pregnant or breastfeeding.

7. **What is "acceptable" in your community regarding women having sex while she is pregnant? Please explain.**

Possible probing topics:

- *KEY PROBE*: What is "acceptable" when the woman is close to delivery? Please explain.
- *KEY PROBE*: What is "acceptable" during breastfeeding? Please explain.
- *KEY PROBE:* How do sex practices in your community change throughout and after pregnancy? Please explain differences from before, during (including close to delivery), and after delivery (i.e. while breastfeeding).
  - How soon after delivery does sexual activity resume?
- How acceptable is it for men to have other sex partners while their partners are P or BF?
  - What about after delivery?

8. **How are women in your community supposed to prepare their vaginas/birth canals for delivery? Why?**

<u>*Possible probing topics:*</u>

- How are women supposed to clean their vaginas while pregnant or breastfeeding?
- What products are supposed to be used every day versus on occasion?
    - How are these used (inserted into vagina, swallowed, etc.)
- Are women supposed to take or use different products for different times throughout your pregnancy or during breastfeeding? Please explain.

D. Ring and PrEP Discussion

Purpose: 1.) To understand what mothers/mothers-in-law *know and think about their daughters/daughters-in-law using oral PrEP or the vaginal ring for HIV prevention while pregnant, and 2.) To hear suggestions about how to get mothers/mothers-in-law support to encourage pregnant or breastfeeding women to use these products.*

As explained before, we are interested in getting your opinion about two different products that women can use for HIV prevention, daily oral PrEP tablets and the monthly vaginal ring. [*Give information on products and/or show or provide samples of products to the group*]

9. **Let's start by talking about daily oral PrEP tablets. What is your first thought when you think about your daughter/daughter-in-law taking oral PrEP to prevent HIV while pregnant?**

<u>*Possible probing topics:*</u>

- *KEY PROBE*: What about while breastfeeding?
- *KEY PROBE*: Would oral PrEP be something you would support your daughter/daughter-in-law using while P or BF? Why or why not
- *KEY PROBE*: What worries do you have about oral PrEP and why? (about your daughter/daughter-in-law's health, your grandchild's health, the father of the child's health, stigma of taking a daily tablet/ARV, etc.)

10. **Depending on the culture, it may be permitted or taboo to take bitter medicine while pregnant. How would this interfere with women in your community's ability to take oral PrEP while pregnant?**

<u>*Possible probing topics:*</u>

- *KEY PROBE*: What about while breastfeeding?
- What other local taboos or practices would make taking oral PrEP while pregnant difficult?

11. **Now let's talk about the vaginal ring. What is your first thought when you think about your daughter/daughter-in-law using a vaginal ring to prevent HIV while pregnant?**

<u>*Possible probing topics:*</u>

- *KEY PROBE*: What about while breastfeeding?
- *KEY PROBE*: Would the vaginal ring be something you would support your daughter/daughter-in-law using during P or BF? Why/why not?
- *KEY PROBE*: What worries do you have and why? (about your daughter's health, your grandchild's health, etc.)

12. **Depending on the culture, it may be permitted or taboo to insert things in the vagina while pregnant. How would this interfere with women in your community's ability to use the vaginal ring while pregnant?**

<u>*Possible probing topics:*</u>

- *KEY PROBE*: What about while breastfeeding?
- What other local taboos or practices would make using the ring while pregnant difficult?

**13. Would mothers/mothers-in-law in your community support their daughters/daughters-in-law using products like these while pregnant? Why or why not?**<br>_Possible probing topics:_<br>• *KEY PROBE*: What about while breastfeeding?<br>• *KEY PROBE*: What preferences would mothers/mothers-in-law have for their daughters/daughters-in-law using oral PrEP versus the ring?<br>• *KEY PROBE*: Would mothers/mothers-in-law be supportive of these products for their daughters/daughters-in-law if they are about as effective as condoms? Please explain.<br>• How would using these products clash with your cultural practices and beliefs around P & BF?<br>o What would help you overcome these clashes with your cultural beliefs or practices?
**14. What do you think is the most important factor that would motivate mothers/mothers-in law to support their pregnant or breastfeeding daughters/daughters-in-law to use the ring or oral PrEP for HIV prevention?**<br>_Possible probing topics:_<br>• *KEY PROBE*: What could be done to help facilitate or encourage women to use these products?<br>• Aside from the woman, who should be involved in her decision to use one of these products while pregnant or breastfeeding? Please explain.<br>• How do you suggest we remove the barriers mothers/mothers-in-law may experience in supporting their daughters/daughters-in-law using these products?
**15. What suggestions do you have on how we can recruit pregnant and breastfeeding women for a future study in which they will try these products?**<br>_Possible probing topics:_<br>• *KEY PROBE*: What do you think about the information we shared about the two products earlier? [*Show product information again if necessary]*<br>o What can we change or add so people who have never heard of oral PrEP or the vaginal ring for HIV prevention understand the products?<br>• What kind of messages would appeal to P and BF women?<br>o How could we gain their trust so women will be willing to join these studies?
E. Wrap Up
**16. Thank you for taking the time to share your opinions with us today. We truly appreciate your willingness to participate and discuss your thoughts and ideas with us. We've now reached the end of our discussion. Do you have any additional comments about pregnancy and breastfeeding practices?**
**17. Do you have any additional comments about oral PrEP or the vaginal ring?**

____
Staff Initials / Date
